# Conservative management without routine bailout stent for non-flow-limiting dissection after DCB angioplasty: a prospective three-arm study

**DOI:** 10.3389/fcvm.2026.1821342

**Published:** 2026-05-21

**Authors:** Jie Chen, Dewen Zhu, Hailiang Ma, Yuanben Lu, Zhenhua Jiang

**Affiliations:** Department of Cardiology, Shaoxing Central Hospital, Shaoxing, Zhejiang, China

**Keywords:** bailout stent, conservative management, coronary dissection, drug-coated balloon, major adverse cardiovascular events (MACE)

## Abstract

The optimal management of non-flow-limiting coronary dissection after drug-coated balloon (DCB) angioplasty remains controversial, particularly in Asian populations. We conducted a prospective, three-arm cohort study to evaluate whether conservative management of non-flow-limiting dissection is non-inferior to bailout stent in patients with *de novo* medium-to-large coronary artery lesions. A total of 276 patients treated with DCB at Shaoxing Central Hospital were enrolled and divided into three Arms: conservative management for non-flow-limiting dissection (Arm 1, *n* = 91), bailout stent for dissection (Arm 2, *n* = 93), and none dissection (Arm 3, *n* = 82). The primary endpoint was 12-month major adverse cardiovascular events (MACE), including cardiac death, target vessel myocardial infarction, and clinically driven target lesion revascularization. Secondary endpoints included BARC 3–5 bleeding, all-cause death, recurrent angina, and ischemic stroke. At 12-month follow-up, the incidence of MACE was 7.7% (7/91) in the conservative management arm, 6.4% (6/93) in the bailout stent arm, and 5.4% (5/92) in the no-dissection control arm (Log-rank *P* > 0.05). The absolute risk difference (ARD) between conservative and bailout stent arms was 1.3% (95% CI: −4.2% to 6.8%). Since the upper 95% CI limit (6.8%) was below the prespecified 10% non-inferiority margin, non-inferiority was confirmed. The corresponding HR was 1.13 (95% CI: 0.58–2.19, *P* > 0.05). No significant between-group differences were observed in any secondary clinical endpoints (all *P* > 0.05). Multivariable Cox proportional-hazards regression analysis demonstrated that dissection management strategy was not an independent predictor of 12-month MACE (adjusted HR 1.12, 95% CI: 0.38–2.21, *P* > 0.05). Prespecified subgroup and sensitivity analyses yielded consistent results with no significant interaction effects (all *P* > 0.05). In conclusion, conservative management of non-flow-limiting NHLBI type A–C dissection after DCB angioplasty for *de novo* medium-to-large coronary lesions is non-inferior to bailout stenting for 12-month MACE in an Asian population, with clinical outcomes comparable to those of patients without dissection. Routine bailout stenting may be considered optional in this clinical setting. These findings suggest the potential value of conservative management as an alternative DCB-only strategy, within the pilot and exploratory nature of this study.

## Introduction

Drug-coated balloon (DCB) angioplasty has emerged as a cornerstone of percutaneous coronary intervention (PCI) in contemporary clinical practice, particularly for the management of *de novo* native coronary lesions, in-stent re-stenosis, and small-vessel disease. Unlike drug-eluting stents (DES), the DCB-only strategy avoids permanent metallic implant coverage, thereby preserving the natural vascular anatomy and compliance, reducing the long-term risks of in-stent re-stenosis, late stent thrombosis, and target lesion re-vascularization (1–4). Additionally, this approach may allow for a shorter duration of dual anti-platelet therapy (DAPT), which is particularly beneficial for patients at high bleeding risk, aligning with the global trend toward precision and patient-centered coronary intervention (4, 5). Despite these well-established advantages, coronary artery dissection remains a common procedural complication during DCB treatment, occurring in approximately 10%–25% of cases depending on lesion complexity, vessel size, and balloon dilation parameters. This complication creates a frequent and clinically challenging dilemma for interventional cardiologists: whether to pursue routine bailout stent implantation or adopt a conservative management strategy for non-flow-limiting dissections (6, 7).

Non-flow-limiting coronary dissections, especially those classified as NHLBI type A–C, are frequently observed after optimal vessel preparation and DCB dilation. These dissections are typically characterized by minimal luminal narrowing (<50%), absence of hemodynamic instability, and no evidence of myocardial ischemia on intraprocedural imaging or electrocardiography (8, 9). Historically, interventionalists have often favored bailout stent implantation for such dissections out of concern for potential acute vessel closure, dissection progression, late re-stenosis, or thrombotic events. However, this prophylactic stent approach is not supported by high-level clinical evidence and may expose patients to unnecessary procedural risks, including contrast-induced nephropathy, vascular access complications, and prolonged DAPT related bleeding. Furthermore, stent implantation in this setting introduces permanent metallic caging, which may disrupt normal vascular physiology, impair endothelial function, and increase the risk of long-term stent-related complications without providing proven incremental clinical benefit (10–13).

In recent years, several observational and prospective studies have challenged the routine use of bailout stent for non-flow-limiting dissection after DCB angioplasty, suggesting that conservative management may be safe and feasible. For example, a prospective study by Cortese evaluated 126 patients with non-flow-limiting dissection after DCB and reported a low rate of adverse events with conservative management, but this study lacked a direct comparison with bailout stent, leaving unresolved the critical question of whether routine stent implantation offers any additional benefit (14). Similarly, a more recent prospective study by Gitto et al. confirmed the safety of conservative management in a Western cohort, but the study was limited by a two-arm design (conservative vs. historical control) and did not include a bailout stent arm (8). Moreover, most prior investigations have enrolled predominantly Western or Japanese populations, and the generalization of their conclusions to Asian populations remains uncertain due to potential differences in lesion morphology, vessel size, comorbidity burden (such as higher rates of diabetes mellitus and hypertension), and vascular healing characteristics. Additionally, few studies have included a no-dissection control arm, which is essential to clarify the true prognostic impact of non-flow-limiting dissection itself and its management strategies.

These evidence gaps highlight the need for a well-designed prospective study to directly compare conservative management and bailout stent for non-flow-limiting dissection after DCB angioplasty, particularly in an Asian population with medium-to-large coronary lesions. Medium-to-large coronary vessels (2.5–4.0 mm) represent the common target for DCB treatment in routine practice, and the management of dissection in these vessels is associated with greater clinical uncertainty due to their role in myocardial perfusion and the potential consequences of dissection progression. Accordingly, we designed a prospective three-arm cohort study to address these critical limitations in the existing literature. The primary objective of this study was to determine whether conservative management of non-flow-limiting NHLBI type A–C dissection after DCB angioplasty is non-inferior to bailout stent with respect to 12-month MACE. Secondary objectives included the evaluation of safety endpoints (including bleeding, all-cause death, and ischemic stroke), the consistency of results across pre-specified sub-Arms, and the robustness of findings through sensitivity analyses. We hypothesized that conservative management would be non-inferior to bailout stent and would yield similar clinical outcomes to patients without coronary dissection, thereby supporting the widespread adoption of a DCB-only strategy without routine prophylactic bailout stent.

This study aims to provide population-specific, high-quality prospective evidence to guide clinical decision-making for the management of DCB-related coronary dissection in routine clinical practice. By directly comparing the two most common management strategies and including a no-dissection control Arm, our findings will help resolve longstanding clinical controversy, fill important evidence gaps, and contribute to the refinement of clinical guidelines for PCI in Asian patients with *de novo* medium-to-large coronary lesions (15, 16).

Although the definitive benefit of DCB-only PCI without stenting remains to be fully demonstrated, conservative management is justified in this study only for non-flow-limiting, hemodynamically stable NHLBI type A–C dissections. These dissections are characterized by minimal luminal narrowing, preserved coronary flow, and absence of intraprocedural ischemia, which have been associated with a low risk of adverse events without routine bailout stenting (14, 17, 18).The rationale is not to neglect potentially high-risk lesions, but to avoid unnecessary stent implantation in anatomically benign and low-risk scenarios, thereby reducing stent-related complications while maintaining equivalent clinical outcomes.

## Methods

This prospective three-arm cohort study was conducted at Shaoxing Central Hospital between January 2022 and December 2024, in accordance with the Declaration of Helsinki and Good Clinical Practice guidelines. The study protocol was approved by the Institutional Review Board (IRB) of Shaoxing Central Hospital (Approval No: SCH-20220528), and written informed consent was obtained from all patients prior to enrollment.

### Study population

Eligible patients were consecutive adults (≥18 years old) who underwent DCB angioplasty for *de novo* medium-to-large coronary artery lesions (vessel diameter 2.5–4.0 mm) confirmed by coronary angiography. The key inclusion criteria were: (1) angiographically documented *de novo* coronary artery stenosis ≥70% in a native coronary vessel with a reference diameter of 2.5–4.0 mm; (2) planned DCB angioplasty as the primary interventional strategy; (3) ability to comply with study follow-up procedures and dual antiplatelet therapy (DAPT) for at least 6 months; and (4) willingness to provide written informed consent.

Exclusion criteria included: (1) acute myocardial infarction (AMI) within 24 h; (2) left main coronary artery disease; (3) severe comorbidities, including end-stage heart failure;New York Heart Association class IV; severe liver or renal dysfunction (estimated glomerular filtration rate <30 mL/min/1.73m^2^, liver enzymes >3 times the upper limit of normal), malignant tumors, or life expectancy <1 year; (4) history of coronary artery bypass grafting or prior PCI; (5) contraindication to DCB, paclitaxel, or anti-platelet therapy; (6) pregnancy or lactation; (7) inability to undergo coronary angiography or follow-up assessments due to physical or cognitive limitations.

### Sample size calculation

Sample size was calculated for a one-sided non-inferiority test of the primary endpoint (12-month MACE incidence). The prespecified absolute risk difference (ARD) non-inferiority margin was set at 10%, which was justified by both clinical and statistical considerations: (1) the expected 12-month MACE rate was low (7%–8%) in both bailout stent and control arms; (2) a 10% margin is widely accepted in DCB-focused coronary trials to avoid an unrealistically large sample size; (3) this threshold represents a clinically acceptable difference when balancing the benefits of avoiding unnecessary stent implantation against potential ischemic risk.

Other prespecified parameters included one-sided *α* = 0.05 and power (1 − *β*) = 85%. Considering a 10% anticipated loss to follow-up, 100 patients per arm (300 total) were required. Calculations were performed using PASS 20.0 and R 4.3.1.

Non-inferiority was confirmed if the upper bound of the two-sided 95% CI for the ARD was below 10%.

### Design and Arm assignment

This was a prospective, observational three-arm cohort study. Randomization was not performed because the decision between conservative management and bailout stenting depended on real-time procedural findings and operator clinical judgment, which is consistent with routine clinical practice.

Patients with non-flow-limiting NHLBI type A–C dissection after DCB angioplasty were allocated to Arm 1 (conservative management) or Arm 2 (bailout stent) based on the treating interventional cardiologist's clinical judgment and dissection morphology. Arm 3 (no-dissection control) was established using 1:1:1 propensity score matching (PSM) to balance baseline demographic, clinical, angiographic, and procedural characteristics with Arms 1 and 2.

Coronary dissection was assessed and classified immediately after DCB dilation by two experienced interventional cardiologists (with >10 years of PCI experience) using coronary angiography according to the NHLBI classification system. Non-flow-limiting dissection was defined as NHLBI type A–C, characterized by minimal luminal narrowing (<50%), absence of hemodynamic instability, and no evidence of myocardial ischemia on intraprocedural electrocardiography. Dissections classified as NHLBI type D–F (flow-limiting) were excluded from the study, as these typically require mandatory bailout stenting and do not represent the clinical dilemma addressed by the present investigation (18, 19). In cases of disagreement regarding dissection classification, a third independent interventional cardiologist was consulted to reach a consensus.

Bailout stenting (Arm 2) was performed for non-flow-limiting dissections with unfavorable morphological features, including dissection length >5 mm, large dissection flap, persistent angiographic haziness, suboptimal vessel expansion, or concern for potential flow disturbance. Conversely, patients in Arm 1 received no additional percutaneous intervention following DCB dilation, and patients in Arm 3 had no angiographically visible dissection after DCB angioplasty and received standard post-PCI management without additional intervention.

All DCBs used were paclitaxel-coated coronary balloon catheters, including commercial products Vesselin DCB and YDB Medical DCB.

### Endpoints

The primary endpoint was 12-month MACE, defined as a composite of cardiac death, target vessel myocardial infarction (TVMI), and clinically driven target lesion re-vascularization (CD-TLR). Cardiac death was defined as death due to cardiac causes (including AMI, heart failure, arrhythmia, or cardiac arrest) or unknown causes without confirmed non-cardiac etiology. TVMI was defined as troponin elevation ≥99th percentile upper reference limit accompanied by ischemic symptoms, new ischemic electrocardiograph changes (ST-segment elevation or depression), pathological Q waves, or imaging evidence of new myocardial loss attributed to the target lesion. CD-TLR was defined as repeated revascularization (PCI or coronary artery bypass grafting) of the target lesion (including ±5 mm of the treated segment) prompted by clinical symptoms of myocardial ischemia or objective evidence of myocardial ischemia (including stress testing or coronary angiography showing ≥50% stenosis in the target lesion) (20).

Secondary endpoints evaluated at 1, 3, 6, 9, and 12 months included BARC grade 3–5 bleeding, all-cause death, recurrent angina (Canadian Cardiovascular Society grade ≥2), and ischemic stroke. BARC 3–5 bleeding was defined according to the Bleeding Academic Research Consortium criteria, including clinically relevant non-major bleeding (grade 3), life-threatening bleeding (grade 4), and fatal bleeding (grade 5). Ischemic stroke was defined as focal vascular neurological deficit persisting for ≥24 h or leading to death, confirmed by cranial computed tomography or magnetic resonance imaging (20).

Endpoint events were adjudicated by an independent endpoint adjudication committee consisting of two cardiologists and one neurologist who were blinded to the study group assignment. The Independent Endpoint Adjudication Committee (IEAC) reviewed all clinical, laboratory, and imaging data related to potential endpoint events to confirm the diagnosis and classify the event according to the study protocol. In cases of disagreement, a fourth independent specialist was consulted to reach a consensus.

### Follow-up protocol

All patients were scheduled for follow-up evaluations at 1, 3, 6, 9, and 12 months after PCI. At each visit, patients underwent physical examination, 12-lead electrocardiography, and laboratory tests. All patients received standard DAPT (aspirin 100 mg once daily plus clopidogrel 75 mg once daily) with a planned 12-month duration. Medication adherence was assessed at each follow-up by self-report and medication history; non-adherence was defined as discontinuation or interruption of either antiplatelet agent for more than 7 days without physician instruction. Additional evaluations were performed as clinically indicated, and telephone follow-up was used to minimize loss to follow-up, with a follow-up rate ≥95%.

### Statistical analysis

All analyses were performed with SPSS 29.0. A one-sided *α* = 0.05 was used for non-inferiority testing, and two-sided tests were employed for all other comparisons with significance set at *α* = 0.05. Continuous variables were summarized as mean ± SD or median (IQR) and compared between Arms using ANOVA or Kruskal–Wallis *H* tests as appropriate. Categorical variables were presented as frequencies (percentages) and compared using *χ*^2^ or Fisher's exact tests.

The primary non-inferiority outcome was 12-month MACE compared between treatment Arms in the ITT population, with a pre-specified non-inferiority margin of 10%. Kaplan–Meier curves and log-rank tests were used to estimate MACE-free survival. Secondary clinical endpoints were analyzed using analogous parametric or nonparametric methods. Multivariable Cox proportional-hazards regression was performed to identify independent predictors of 12-month MACE. Pre-specified sub-Arm analyses were conducted using interaction terms, and sensitivity analyses including the PP population, IPTW and complete-case analysis were performed to assess the robustness of the results.

## Result

**Figure 1:**
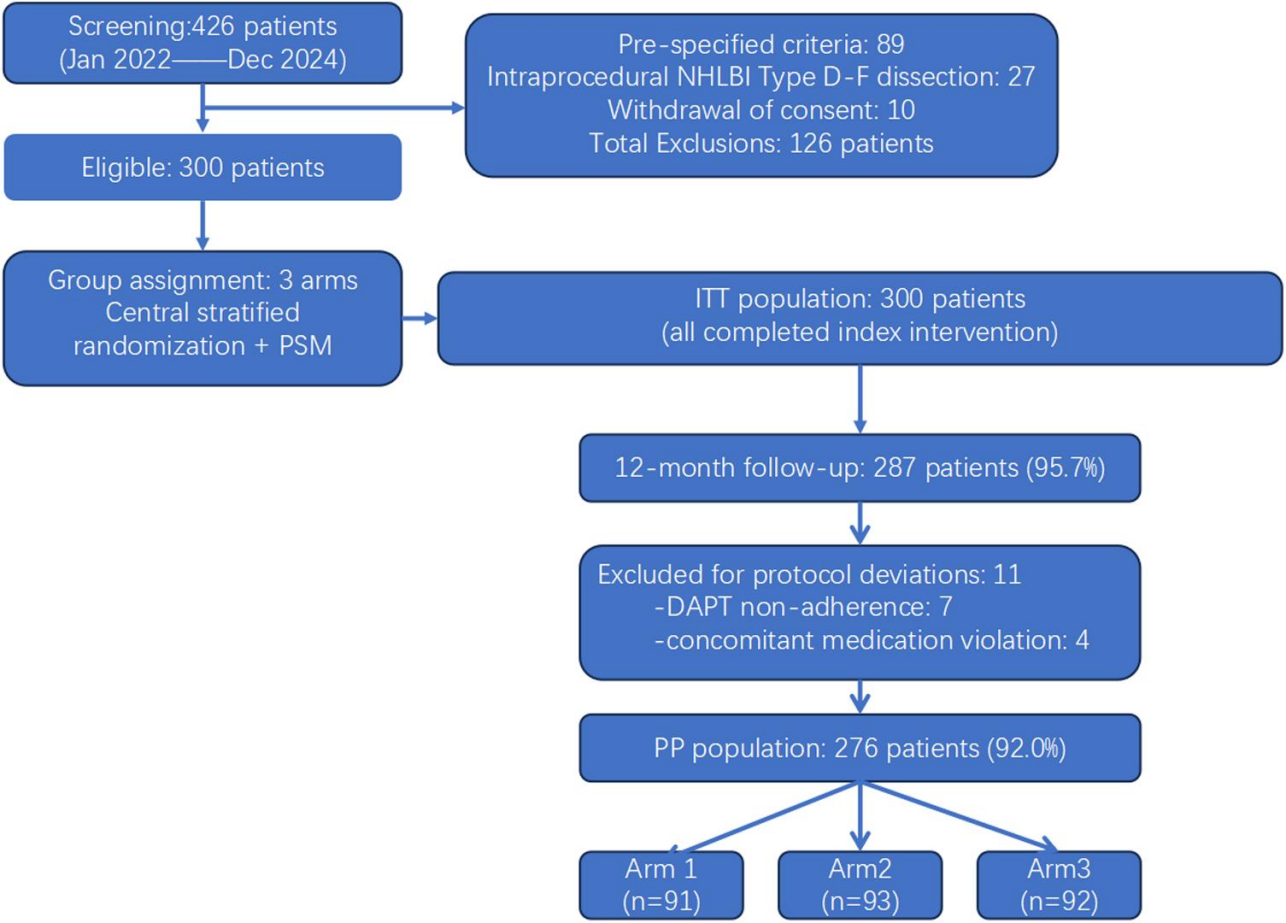
CONSORT-style study enrollment and patient flow diagram.

The patient enrollment and study flow are illustrated in Figure 1. A total of 426 patients undergoing elective DCB angioplasty for *de novo* coronary lesions (reference vessel diameter 2.5–4.0 mm) were screened between January 2022 and December 2024. Of these, 126 patients were excluded due to prespecified exclusion criteria (*n* = 89), intraprocedural NHLBI type D–F flow-limiting dissection (*n* = 27), and voluntary withdrawal of consent (*n* = 10). The remaining 300 eligible patients were assigned to three study arms (100 per arm): patients with NHLBI type A–C non-flow-limiting dissection were allocated to Arm 1 (conservative management) or Arm 2 (bailout stent) based on operator clinical judgment; Arm 3 (no-dissection control) was constructed using 1:1:1 propensity score matching (PSM) to balance baseline characteristics. All 300 patients were included in the intention-to-treat (ITT) population. After excluding 11 patients with major protocol deviations (DAPT non-adherence *n* = 7; concomitant medication violation *n* = 4), 276 patients (92.0%) constituted the per-protocol (PP) population.

### Baseline and procedural characteristics

No statistically significant differences were observed in all baseline demographic, clinical, biochemical, electrocardiograph, angiographic and procedural characteristics across the three study arms (all *P* > 0.05; Tables 1 and 2).

**Table 1:** Baseline characteristics of the study arms.

Characteristic	Arm 1: (*n* = 91)	Arm 2: (*n* = 93)	Arm 3: (*n* = 92)	*P*-value
Demographics				
Age, years	62.6 ± 8.7	62.3 ± 9.6	63.2 ± 9.5	0.802
Male sex, *n* (%)	74 (81.3)	69 (74.2)	77 (83.7)	0.247
BMI, kg/m^2^	25.9 ± 3.2	25.5 ± 2.8	25.4 ± 3.4	0.617
Risk factors, *n* (%)				
Hypertension	79 (86.8)	72 (77.4)	74 (80.4)	0.246
Diabetes mellitus	42 (46.2)	35 (37.6)	39 (42.4)	0.502
Current smoking	39 (42.9)	31 (33.3)	45 (48.9)	0.095
Prior myocardial infarction	9 (9.9)	8 (8.6)	10 (10.9)	0.873
Biochemical indicators				
TG, mmol/L	1.95 ± 0.30	1.90 ± 0.30	1.92 ± 0.31	0.545
TC, mmol/L	4.23 ± 0.82	4.39 ± 0.87	4.52 ± 1.04	0.103
HDL-C, mmol/L	1.18 ± 0.21	1.18 ± 0.20	1.14 ± 0.21	0.266
LDL-C, mmol/L	2.33 ± 0.40	2.27 ± 0.48	2.29 ± 0.44	0.563
Scr, µmol/L	66.4 ± 12.2	62.8 ± 11.6	65.2 ± 11.5	0.106
BNP, ng/mL	45.4 ± 14.7	46.3 ± 14.6	47.8 ± 15.9	0.787
Echocardiograph				
LVEF, %	60.9 ± 3.6	61.8 ± 3.3	61.6 ± 3.2	0.232
LVESD, mm	36.1 ± 1.8	35.7 ± 1.8	35.9 ± 1.7	0.294
LVEDD, mm	52.8 ± 1.8	52.4 ± 2.7	52.5 ± 1.7	0.478
DAPT duration, months	11.8 ± 1.3	11.9 ± 1.2	11.7 ± 1.4	0.762

BMI, body mass index; TG, triglyceride; TC, total cholesterol; HDL-C, high-density lipoprotein cholesterol; LDL-C, low-density lipoprotein cholesterol; Scr, serum creatinine; BNP, brain natriuretic peptide; LVEF, left ventricular ejection fraction; LVESD, left ventricular end-systolic diameter; LVEDD, left ventricular end-diastolic diameter.

**Table 2:** Angiographic and procedural characteristics of the three study arms.

Characteristic	Arm 1: (*n* = 91)	Arm 2: (*n* = 93)	Arm 3: (*n* = 92)	*P*-value
Target vessel, *n* (%)				0.815
Left anterior descending artery	37 (40.7)	34 (36.6)	40 (43.5)	
Right coronary artery	37 (40.7)	39 (41.9)	38 (41.3)	
Left circumflex artery	17 (18.7)	20 (21.5)	14 (15.2)	
Lesion location, *n* (%)				0.419
Proximal lesion	30 (33.0)	32 (34.4)	27 (29.3)	
Mid lesion	52 (57.1)	49 (52.7)	47 (51.1)	
Distal lesion	9 (9.9)	12 (12.9)	18 (19.6)	
Calcification	30 (33.0)	20 (21.5)	32 (34.8)	0.101
Bifurcation	20 (22.0)	17 (18.3)	19 (20.7)	0.819
Reference vessel diameter (mm)	3.22 ± 0.18	3.20 ± 0.18	3.20 ± 0.17	0.769
Lesion length, (mm)	14.6 ± 1.9	14.1 ± 2.0	14.0 ± 1.9	0.095
Pre-procedural diameter stenosis, %	87.1 ± 2.7	86.9 ± 3.0	87.2 ± 2.5	0.812
Pre-dilation strategy, *n* (%)				
Semi-compliant balloon	75 (82.4)	64 (68.8)	74 (80.4)	0.059
Non-compliant balloon	18 (19.8)	17 (18.3)	29 (31.5)	0.066
Cutting balloon	22 (24.2)	25 (26.9)	21 (22.8)	0.808
Pre-dilation balloon diameter, mm	2.61 ± 0.27	2.50 ± 0.31	2.53 ± 0.25	0.075
Pre-dilation max pressure, atm	10.2 ± 0.6	10.0 ± 0.8	10.0 ± 0.7	0.318
DCB specifications				
DCB diameter, mm	3.01 ± 0.39	3.02 ± 0.42	2.95 ± 0.41	0.392
DCB length, mm	15.4 ± 2.7	15.1 ± 2.9	15.2 ± 2.8	0.422
DCB inflation pressure, atm	6.68 ± 0.31	6.70 ± 0.27	6.72 ± 0.28	0.606
DCB inflation duration, s	65.8 ± 3.2	65.4 ± 3.1	65.0 ± 3.0	0.227

Baseline cardiovascular risk factors, clinical presentation, left ventricular function parameters and routine biochemical indicators were well-matched among groups. Target vessel distribution, lesion location and morphological features, as well as quantitative coronary angiography (QCA) metrics of the target lesions, were homogeneous across arms. Procedural strategies were strictly standardized, with consistent pre-dilation approaches, DCB implantation parameters and adherence to study protocol across all groups; bailout stent in Arm 2 was uniformly performed with second-generation DES as predefined.

The median actual DAPT duration was 12.0 months across all groups, with no significant between-group difference. Overall DAPT adherence was satisfactory, with 7 patients (2.3%) experiencing protocol-defined non-adherence.

### 3.3 Primary endpoint results

**Figure 2:**
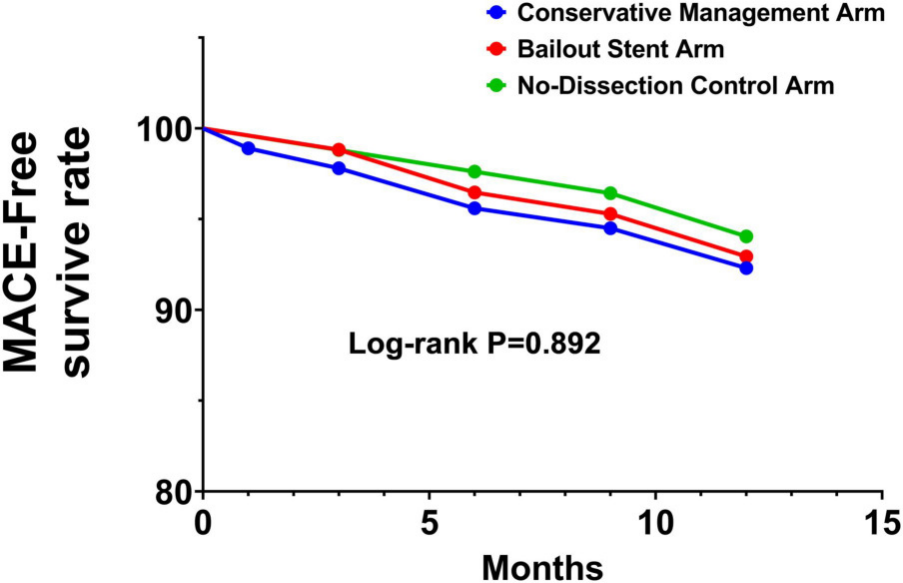
Twelve-Month MACE-free survival.

At 12-month follow-up, the incidence of MACE was 7.7% (7/91) in the conservative management arm, 6.4% (6/93) in the bailout stent arm, and 5.4% (5/92) in the no-dissection control arm (Log-rank *P* > 0.05). The absolute risk difference (ARD) between conservative and bailout stent arms was 1.3% (95% CI: −4.2% to 6.8%). Since the upper 95% CI limit (6.8%) was below the prespecified 10% non-inferiority margin, non-inferiority was confirmed. The corresponding HR was 1.13 (95% CI: 0.58–2.19, *P* > 0.05). Kaplan–Meier curves showed highly overlapping MACE-free survival throughout follow-up, with no significant between-group difference (Log-rank *P* > 0.05), indicating comparable 12-month clinical outcomes with different management strategies for non-flow-limiting dissection (Figure 2).

**Figure 3:**
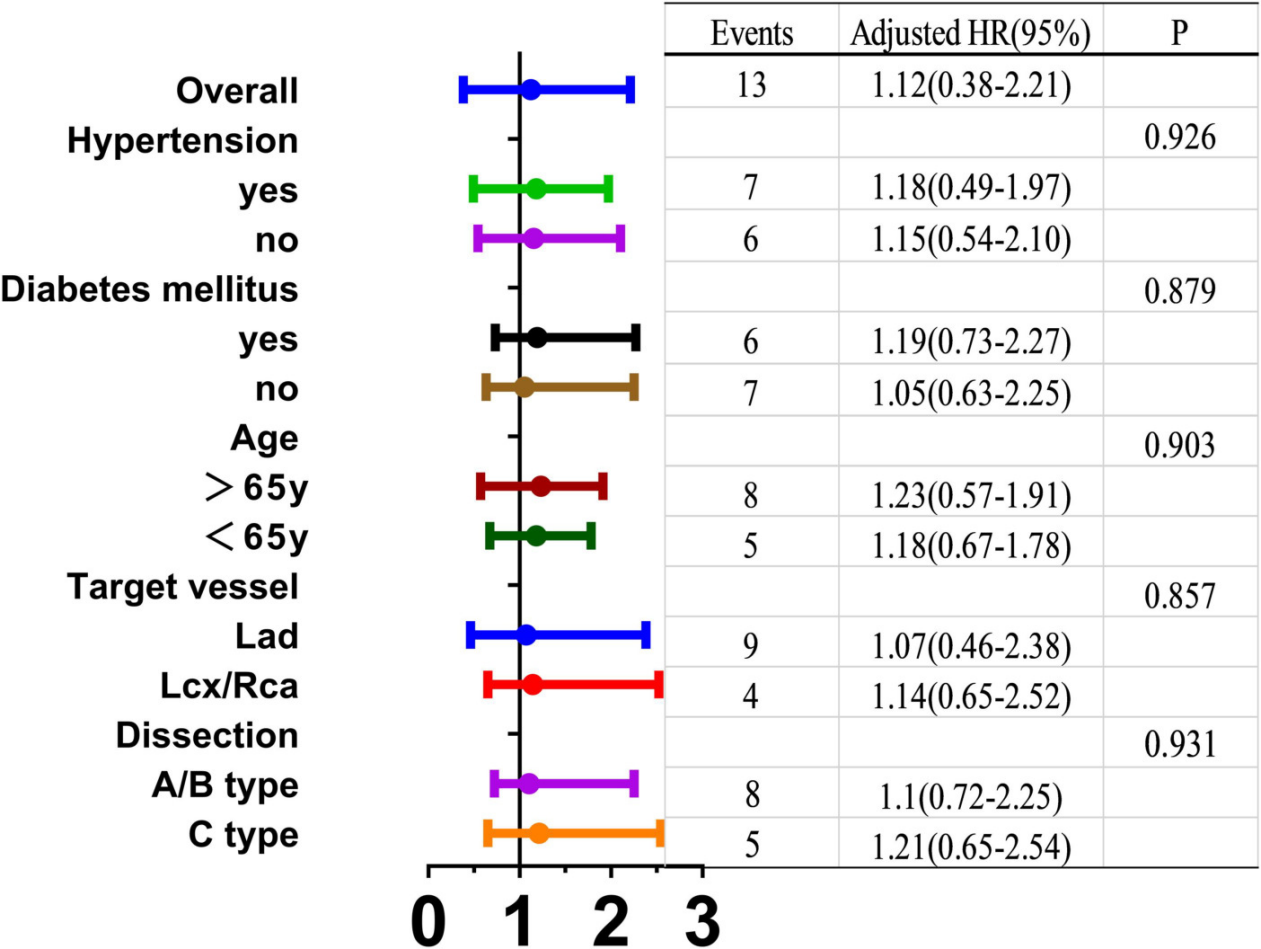
Cox proportional hazards regression analysis.

Cox proportional hazards regression analysis (Figure 3) was performed to identify independent predictors of 12-month MACE in the PP population, adjusted for potential con-founders including age, hypertension, diabetes mellitus, Target vessel, and dissection sub-type (A/B vs. C). After multi variable adjustment, the management strategy for NHLBI type A–C non-flow-limiting dissection (conservative management vs. bailout stent) was not an independent predictor of 12-month MACE (*P* > 0.05).

### 3.4 Secondary clinical endpoints

There were no significant between-group differences in any of the prespecified secondary clinical endpoints at 1, 3, 6, 9, and 12 months of follow-up. The incidence of BARC 3–5 grade bleeding, all-cause death, recurrent angina (CCS ≥2), and ischemic stroke was low and comparable across the three study arms (all *P* > 0.05). No episodes of BARC 3–5 bleeding or ischemic stroke were observed in any group during the entire follow-up period. These findings demonstrated consistent safety and clinical outcomes among patients managed with different strategies for non-flow-limiting dissection.

### 3.5 Subgroup analyses

Prespecified subgroup analyses were conducted according to reference vessel diameter, diabetes status, and dissection subtype. In all tested subgroups, the incidence of 12-month MACE in Group 1 remained non-inferior to that in Groups 2 and 3. Tests for interaction revealed no significant effect modification across all subgroups (all *P* > 0.05), confirming that the primary results were consistent and not materially altered by these clinical or lesion characteristics.

### 3.6 Sensitivity analyses

A series of sensitivity analyses were performed to evaluate the robustness of the primary results. Exclusion of patients with type C dissection or those with suboptimal adherence to DAPT yielded similar findings, with no significant between-group differences in 12-month MACE rates (all *P* > 0.05). Moreover, results derived from inverse probability of treatment weighting analysis were highly consistent with the primary analysis.

## Discussion

This prospective three-arm study represents the first investigation focusing on *de novo* medium-to-large coronary artery lesions in an Asian population to directly compare conservative management, bailout stent, and a no-dissection control arm following DCB angioplasty. The key finding of the present analysis was that conservative management of non-flow-limiting NHLBI type A–C dissection after DCB angioplasty was non-inferior to bailout stent with respect to 12-month MACE, and yielded similar clinical outcomes compared with patients without evidence of coronary dissection. Importantly, no excess signal of harm was observed with conservative management. These findings provide preliminary evidence supporting the potential role of a DCB-only strategy without routine bailout stent in patients with *de novo* medium-to-large coronary lesions who develop non-flow-limiting dissections during PCI.

The management of non-flow-limiting coronary dissection after DCB treatment has long been a topic of clinical debate, with uncertainty regarding the need for bailout stent implantation to prevent adverse events. Previous prospective studies, including those by Cortese et al. and Gitto et al., provided preliminary evidence suggesting that conservative management may be safe in selected patients with dissection after DCB (8, 17). However, those studies lacked a direct comparison with bailout stent, leaving unresolved the critical question of whether routine stent implantation offers incremental benefit. The present study addresses this evidence gap by including a bailout stent arm, allowing a head-to-head comparison between the two strategies. Our results demonstrate that conservative management is not inferior to bailout stent, suggesting that routine stent implantation for non-flow-limiting dissection provides no meaningful clinical advantage and may be safely omitted. In this way, our findings extend and strengthen the existing literature by providing a direct comparative evidence base for clinical decision-making. Furthermore, a recent large-scale systematic review and meta-analysis including more than 4,000 patients has provided benchmark event rates for clinical outcomes following DCB angioplasty. These data are consistent with our findings and further support that routine bailout stent implantation provides no incremental clinical benefit and may be safely omitted in patients with non-flow-limiting coronary dissection (1).

Compared with studies conducted in European, American, and Japanese populations, the present investigation provides population-specific data for Chinese patients with *de novo* medium-to-large coronary lesions (10–13). Most prior studies enrolled predominantly Western or Japanese cohorts, and the generalizability of their conclusions to Asian populations has remained uncertain due to potential differences in lesion morphology, vessel size, comorbidity burden, and healing characteristics. Our results confirm that the safety of conservative management observed in other cohorts is reproducible in an Asian patient population, supporting the broader application of a DCB-only strategy beyond previously studied populations. By focusing on medium-to-large vessels (2.5–4.0 mm), which represent the most common and clinically relevant scenario for DCB treatment in routine practice, the present findings are particularly applicable to real-world interventional cardiology practice.

The favorable clinical outcomes observed with conservative management can be understood through several complementary pathophysiological mechanisms. First, paclitaxel released from the DCB exerts potent inhibitory effects on smooth muscle cell proliferation and migration, while promoting reendothelialization and vascular healing. These biological effects facilitate the closure and re-reendothelialization of intimal dissection flaps, reducing the risks of thrombus formation, flow disturbance, and restenosis. Second, non-flow-limiting dissections classified as NHLBI type A–C are typically limited to the intimal layer without extensive medial injury, large intramural hematoma, or anatomical features associated with unstable flow dynamics. Such dissections have a high propensity for spontaneous healing without mechanical support from a stent. Third, bailout stent implantation, although anatomically intuitive, introduces permanent metallic caging, which may disrupt vascular physiology, impair endothelial function, and increase the long-term risks of in-stent restenosis and late stent thrombosis (17, 21–23). In the absence of demonstrated benefit, routine stent for benign dissection morphology exposes patients to the risks of stent-related complications without justification. Collectively, these mechanisms provide a strong biological rationale for avoiding unnecessary stent implantation in this specific clinical setting.

The findings of the present study carry meaningful and actionable clinical implications for daily interventional practice. Most importantly, they support a conservative strategy for non-flow-limiting dissection after DCB and strongly discourage routine bailout stent. This approach reinforces the value of the DCB-only concept, which aims to achieve effective lesion preparation and drug delivery without leaving behind a permanent metallic implant. Avoiding unnecessary stent implantation reduces procedural costs, simplifies post-procedural management, and may allow for a shorter duration of DAPT, which is especially relevant for patients at elevated bleeding risk (5). From a public health and guideline perspective, this study provides prospective, population-specific evidence for Asian patients, addressing an important gap in the literature that has limited guideline certainty in this region. By validating the safety of conservative management in a real-world Asian cohort, our results help to refine treatment algorithms and promote more precise, patient-centered coronary intervention (24).

Intracoronary imaging could significantly refine patient selection for conservative management. OCT or IVUS allows precise evaluation of dissection depth, flap mobility, residual lumen area, and intramural hematoma, which are critical for identifying truly low-risk dissections that can be safely managed without stenting. Routine imaging would help operators better stratify risk, avoid unnecessary bailout stenting for genuinely benign lesions, and promptly intervene in morphologically high-risk cases that are occult on angiography. Future studies incorporating intracoronary imaging are warranted to establish standardized selection criteria for conservative management after DCB angioplasty.

Several important strengths of the present study deserve emphasis. First, the three-arm prospective design allows for direct and reliable comparison of conservative treatment, bailout stent, and a no-dissection control group, providing clarity that previous two-arm or retrospective studies could not achieve. Second, rigorous methodology, including propensity score matching to reduce baseline confounding, independent endpoint adjudication, and a high follow-up rate, enhances the internal validity and credibility of the findings. Third, the study population was carefully selected to reflect a clinically relevant cohort with *de novo* medium-to-large coronary lesions, the scenario in which DCB treatment is most widely used and clinical uncertainty is greatest. Finally, the study was conducted in a routine clinical setting without highly selected enrollment criteria, improving the translational potential and real-world applicability of the results.

Importantly, conservative management was applied exclusively to low-risk, non-flow-limiting, stable dissections; flow-limiting dissections (NHLBI type D–F) were excluded and managed with mandatory stenting. Our strategy does not assume an established benefit of DCB-only PCI, but evaluates whether de-escalation of treatment (omitting routine bailout stenting) is non-inferior in a well-defined low-risk subgroup (14, 18).

Despite these strengths, several limitations should be acknowledged when interpreting the findings.

First, the allocation between conservative management and bailout stenting was based on operator judgment rather than formal randomization, which may introduce confounding by indication. Operators may have selected morphologically higher-risk dissections for bailout stenting, and unmeasured confounders such as detailed dissection morphology could not be fully adjusted for, even with balanced baseline characteristics.

Second, the follow-up duration was limited to 12 months, which may be insufficient to capture late restenosis, very-late stent thrombosis, or delayed anatomical healing of coronary dissections. Longer-term follow-up of at least 24 months is warranted to better evaluate the durability and long-term safety of a conservative strategy for non-flow-limiting dissection after DCB angioplasty. We plan to continue observing this cohort to obtain extended long-term data (2, 3, 25, 26).

Third, the absolute number of MACE events was low, resulting in wide confidence intervals and limited statistical power. A *post-hoc* power calculation based on observed event rates confirmed insufficient power to detect small but clinically relevant between-group differences. Therefore, the conclusions should be interpreted with appropriate caution.

Fourth, coronary dissection assessment was based solely on coronary angiography without intracoronary imaging (OCT/IVUS). Angiography may underestimate dissection depth, miss intramural hematoma or unstable flap features, and misclassify a substantial proportion of lesions. Intracoronary imaging could more accurately stratify risk and refine patient selection for conservative management (23, 26).

Fifth, the study lacked routine angiographic follow-up at 12 months, so we could not systematically evaluate late lumen loss, binary restenosis, or anatomical healing of the dissections, which limited lesion-level efficacy assessment.

Sixth, lesion-specific efficacy endpoints including target-lesion failure (TLF), target-vessel failure (TVF), residual stenosis, late lumen loss, and binary restenosis were not systematically collected in this study. Therefore, these anatomical and lesion-level outcomes could not be reported. Future investigations are warranted to collect these key metrics to comprehensively evaluate lesion-specific efficacy after DCB angioplasty.

Seventh, this study has a relatively small sample size and should be considered a pilot exploratory investigation. It was not powered to draw definitive conclusions on safety. All findings should be interpreted with appropriate caution.

## Conclusion

In conclusion, this prospective three-arm cohort study demonstrates that conservative management of non-flow-limiting NHLBI type A–C dissection after DCB angioplasty for *de novo* medium-to-large coronary lesions is non-inferior to bailout stenting with respect to 12-month MACE in a Chinese population. Furthermore, clinical outcomes in patients with non-flow-limiting dissection managed conservatively were comparable to those in patients without dissection. Multi-variable Cox regression and prespecified subgroup analyses confirmed that the dissection management strategy was not an independent predictor of adverse events, with consistent results across key patient and lesion subgroups. These findings address a critical evidence gap in Asian populations and challenge the routine use of prophylactic bailout stenting for non-flow-limiting dissection following DCB angioplasty. Our findings suggest that conservative management may represent a reasonable alternative to bailout stenting in this specific setting. Given the pilot design, single-center setting, and relatively small sample size, definitive conclusions regarding safety cannot be drawn.

## Data Availability

The original contributions presented in this study are included in the article/supplementary material, further inquiries can be directed to the corresponding author.
